# Mutational Hotspot of TET2, IDH1, IDH2, SRSF2, SF3B1, KRAS, and NRAS from Human Systemic Mastocytosis Are Not Conserved in Canine Mast Cell Tumors

**DOI:** 10.1371/journal.pone.0142450

**Published:** 2015-11-12

**Authors:** Eleonora Zorzan, Katia Hanssens, Mery Giantin, Mauro Dacasto, Patrice Dubreuil

**Affiliations:** 1 Department of Comparative Biomedicine and Food Science, University of Padua, Legnaro, Padua, Italy; 2 Inserm U1068, Centre de Recherche en Cancérologie de Marseille, Signalisation, Hematopoiesis and Mechanisms of Oncogenesis, Centre de référence des mastocytoses, Institut Paoli Calmettes, CNRS, Aix Marseille Université, Marseille, France; Colorado State University, UNITED STATES

## Abstract

**Introduction:**

Both canine cutaneous mast cell tumor (MCT) and human systemic mastocytosis (SM) are characterized by abnormal proliferation and accumulation of mast cells in tissues and, frequently, by the presence of activating mutations in the receptor tyrosine kinase V-Kit Hardy-Zuckerman 4 Feline Sarcoma Viral Oncogene Homolog (c-KIT), albeit at different incidence (>80% in SM and 10–30% in MCT). In the last few years, it has been discovered that additional mutations in other genes belonging to the methylation system, the splicing machinery and cell signaling, contribute, with c-KIT, to SM pathogenesis and/or phenotype. In the present study, the mutational profile of the Tet methylcytosine dioxygenase 2 (TET2), the isocitrate dehydrogenases 1 and 2 (IDH1 and IDH2), the serine/arginine-rich splicing factor 2 (SRSF2), the splicing factor 3b subunit 1 (SF3B1), the Kirsten rat sarcoma viral oncogene homolog (KRAS) and the neuroblastoma RAS viral oncogene homolog (NRAS), commonly mutated in human myeloid malignancies and mastocytosis, was investigated in canine MCTs.

**Methods:**

Using the Sanger sequencing method, a cohort of 75 DNA samples extracted from MCT biopsies already investigated for c-KIT mutations were screened for the “human-like” hot spot mutations of listed genes.

**Results:**

No mutations were ever identified except for TET2 even if with low frequency (2.7%). In contrast to what is observed in human TET2 no frame-shift mutations were found in MCT samples.

**Conclusion:**

Results obtained in this preliminary study are suggestive of a substantial difference between human SM and canine MCT if we consider some target genes known to be involved in the pathogenesis of human SM.

## Introduction

In dogs, cutaneous mast cell tumor (MCT) is the most common skin tumor, and it accounts for up to 10–30% of all cases. MCTs occur mostly in the dermis and subcutaneous tissue but some visceral forms can also be located in other sites e.g. gastrointestinal tract and spine bone marrow as well as liver, oral cavity, urethra, salivary gland, nasopharynx and spleen [1–3]. It is commonly identified as a solitary neoplastic mass in the skin and/or subcutaneous tissue of older dogs, with a mean age of onset of approximately 9 years of age. Some dog breeds, such as Boxers, Labrador Retrievers and Shar Pei, are more prone to develop MCTs [4,5].

Activating mutations of the tyrosine kinase receptor c-KIT, which binds to stem cell factor (SCF), a known hematopoietic cytokine, have been described in canine MCTs. Mutations in c-KIT occur in 15–50% of MCTs, and have been associated with a more aggressive tumoral phenotype [6], possibly due to an increased proliferation and a resistance to apoptosis [7,8]. The most common type of mutations identified in canine MCTs are internal tandem duplications (ITD) involving exon 11 [6,9] but also deletions and point mutations in exons 8, 9 and 11 can occur [2,10].

Human mastocytosis is a rare and clonal hematopoietic disease described as the proliferation and the accumulation of abnormal mast cells in the bone marrow and organs [11]. Mastocytosis is schematically divided into cutaneous mastocytosis (CM) and systemic mastocytosis (SM). Localized mast cell tumors as mastocytomas and mast cell sarcoma are very rare. CM is usually diagnosed at birth or in childhood and spontaneously regress over time. However, some types are locally invasive, clinically very severe and, consequently, hard to treat. In most adult patients, the disease is systemic, although also the skin is often affected.

Most cases of SM are associated with the presence of activating mutations in the c-KIT proto-oncogene. The most frequent KIT genetic alteration is the substitution of aspartic acid to valine at position 816 (KIT D816V), that leads to the constitutive activation of the kinase domain of the receptor [12].

It has been recently discovered as further cooperating events may contribute to the phenotype and/or the pathogenesis of SM [13,14] e.g. mutations in tet methylcytosine dioxygenase 2 (TET2) which have been reported in 40% of KIT D816V-positive SM cases [15]. The enzyme TET2 regulates gene methylation and expression, catalyzing the conversion of 5-methylcytosine (5-mC) to 5-hydroxymethylcytosine (5-hmC) [16]. In SM, it has recently been reported a more aggressive disease and an overall worse prognosis when there is the coexistence of KIT D816V and TET2 mutations [17]. Other mutations were identified in isocitrate dehydrogenase 1 and 2 (IDH1 and IDH2, respectively). They affect both histone modifications and DNA methylation, catalyzing the decarboxylation of isocitrate to alpha-ketoglutarate (or 2-oxoglutarate, 2-OG). Hotspot mutation sites are represented by heterozygous substitution clusters in conserved arginines R132 of IDH1 and R140 and R172 of IDH2 [18]. Further additional mutations were found in genes encoding for components of the splicing machinery involved in the intron splicing during pre-mRNA maturation, in particular the serine/arginine-rich splicing factor 2 and the splicing factor 3b, subunit 1 (respectively SRSF2 and SF3B1). Overall, recent data are suggestive of a specific hierarchy, where TET2 gene alterations arise in early progenitor cells, while SRSF2 mutation can occur relatively later during the ontogeny but both prior to KIT mutation during the disease progression [11]. Likewise, neuroblastoma RAS Viral (V-Ras) oncogene homolog (NRAS) mutations have also been reported in SM, having the potential to precede KITD816V in clonal development [19].

Besides SM, loss-of-function mutations in TET2 as well as alterations in other genes mentioned above have been also reported in a variety of hematological malignancies, including acute myeloid leukemias (AMLs), chronic myelomonocytic leukemia (CMML), myeloproliferative neoplasms (MPNs), myelodysplastic syndromes (MDS) and lymphoid malignancies [20–24]. To the best of our knowledge, no data on mutational status of these genes are available for canine MCTs.

In the present study, hypothesizing analogies in molecular mechanisms and gene dysfunctions with human SM and hematopoietic diseases, the mutation profile of genes commonly mutated in myeloid malignancies has been evaluated in a cohort of 75 MCTs, most of them previously screened for c-KIT mutations [8].

## Materials and methods

### Samples and ethical statement

All tissue biopsies and blood samples were not specifically taken for the purposes of this study; they were part of authors *in-house* collections and were already used in previous studies [8, 25, 26].

Tissue biopsies were originally collected as part of routine treatment procedures from dogs affected by at least one histologically-confirmed MCT (Patnaik grade II or III) [27], recurrent after surgery (as standard of care) and/or nonresectable. Female and male dogs, regardless of breed, were previously recruited with owner consent from veterinary clinics in France and in United States.

Blood samples were collected in Italy from 39 healthy random-source adult kennel dogs undergoing routine examination as described in details previously [26]. An Institutional Animal Care and Use Committee approval number was not requested because of an agreement between the Faculty of Veterinary Medicine of University of Padua (Italy) and the kennel for the execution of routinary clinical checkups as described in details previously [26]. Animal care was carried out in accordance with good veterinary practices.

### DNA extraction, PCR and sequence analysis

Genomic DNA was extracted from 75 frozen canine MCT tissue samples using QIAamp DNA Mini Kit (Qiagen France, Paris, France), according to manufacturer’s protocol. In the 23% of the cohort samples, different c-KIT mutations were previously identified in exons 8, 9 and 11[8]. Among them, internal tandem duplications of exon 11 represented 36% of total mutations registered.

In the present study, PCR amplifications of all TET2 coding exons and the hot-spot regions of IDH1 (exon 2), IDH2 (exon 1), SF3B1 (exons 13, 14, 15, 16), SRSF2 (exon 1), NRAS (exons 1, 2) and KRAS (exon 1) were executed. Primer oligonucleotide sequences were designed using the AmplifX software (http://crn2m.univ-mrs.fr/AmplifX) and CanFam3.1 genome sequences available http://www.ncbi.nlm.nih.gov/. Primer sequences, are reported in Table 1. For every exon analyzed, the extreme parts of the flanking introns were also sequenced (around 100 bp upstream the 5´-end and downstream its 3´-end) to check for the presence of alternative splicing sites. All the detected variations were analyzed by using the tool Berkeley Drosophila Genome Project (BDGP, http://www.fruitfly.org) that computed splice sites predictions. Genes were amplified using Taq Phire^®^ Hot Start II DNA Polymerase (Thermo Fisher Scientific, Walthman, MA, USA). The reaction mix contained the following reagents: 1X Phire^®^ Reaction Buffer, 200 μM dNTPs, 0.5 μM of each primer and 0.15 μL of the enzyme (in a final volume of 22 μL). Approximately, 30 ng of genomic DNA were added to each PCR reaction and amplified through the following thermal protocol: an initial denaturation step at 98°C for 30 sec, an amplification step of 35–40 cycles (denaturation at 98°C for 5 sec, annealing at the primer-specific temperature for 5 sec and elongation at 72°C for 5–10 sec depending on the length of the PCR product) and a final elongation step at 72°C for 1 min. PCR products were purified and sequenced in an ABI 3730 sequencer. Sequence PCR reactions were performed with both primer forward (F) and reverse (R) using the Big Dye Terminator V1.1. (Applied Biosystem, Life Technologies, Carlsbad, USA) and the mix included: 3.2 pmol of oligo F or R, 1μL of Big Dye Terminator V1.1, 1X reaction buffer and water (in a final volume of 10 μL). The thermal protocol consisted in: an initial denaturation (1 min at 96°C) followed by 25 cycles of 10 sec at 96°C, 5 sec at 50°C and 2 min at 60°C.

**Table 1:** Forward (F) and reverse (R) primer sequences of canine genes included in the present study and used for polymerase chain reaction with the corresponding annealing temperature and product length.

GENE AND PRIMER SEQUENCES (5´-3´)	EXON	TEMP. ANNEALING	PRODUCT LENGHT
**KRAS**			
F: CTCATCTGTGGTCAACTGAA	1	60°C	466 bp
R: AGCCAATGGAACCCAAGTA			
**IDH1**			
F: TGGCACTGTCTTCAGGGAAGCTAT	2	70°C	163 bp
R: TGGGCAACCAAGGACAGGAAAA			
**IDH2**			
F: CTCCATCTCTGTCCTCGTAGAGT	4	67°C	343 bp
R: TTAGCACCGCTGCCATCCTTT			
**NRAS**			
F: TCTCTAGTTGTGGCTCGCCCATTA	1	65°C	223 bp
R:CAAAAGCCAGAGGTAGGGTCAGT			
F:GCTAGGAGCTTATCTAACCTTGGC	2	60°C	367 bp
R: TGCGGTATCCTCATTTCCTGTTCC			
**SF3B1**			
F: ACTGGAGGATCAAGAGCGTCAT	13	67°C	1101 bp
R: GCTGTCGTGTTACGGACATACT			
F: ATGCTAGAGTGGAAGGTCGAGA	14	67°C	855 bp
R: TGTGTTGGCGGATACCCTT			
F: GACCATTAGCGCTTTGGCCATT	15–16	67°C	529 bp
R: GTTCCACAACACTGCTTCACCA			
**TET2**			
F: AGCCTGATGGAACAGGATAGA	3	60°C	782 bp
R: GCCTGACTGTTAATGGCA			
F:CAAGAAAGTAATCCAGGCAAAGGC	3	60°C	718 bp
R: AATACCGTTCAGAGCTGCCA			
F: CCTGTCCCTTCCAGAAACCAGAAA	3	60°C	605 bp
R: TGTTGGGTTATGCTTGAGGTGTTC			
F: CCCCAACCAAAGTAACACAGCTCT	3	60°C	702 bp
R: GCTTTGGATGAAGGGTCTGTCTTG			
F: GGCATCACTGCGGTCAGTTCTT	3	60°C	715 bp
R: ATTCTGTCCTTGCTCCAATCCCA			
F: TCCCAAGGCAACAATGATCAGC	3	60°C	760 bp
R: GGGGTGGAATCTCTTGCTTAGTTG			
F: CTCCCCAGAAGGACATTCAAAAG	3	60°C	784 bp
R: CTCTCTTGCACAGCACAAGCAT			
F: GGATAAGCTTTGTGGATGTAGCCT	4	60°C	371 bp
R: GCTCGCAGACTATTAGTCCTGT			
F: TCCAGTTTGCTTGGCTTAGAC	5	60°C	380 bp
R: GAGCAACGTTCATTTCAACTAGC			
F: AATGCCCTAGTTGTGACCCAG	6	60°C	421 bp
R: AAATGTCGGTTCAACTCCCTTCCC			
F: CCAGAATCCAAGATTGGTAGCC	7	60°C	295 bp
R: GACTGCTTACTTCATCTGTACTCA			
F: TCATTTGGATCTAGGCTGTAGGGG	8	65°C	336 bp
R: AACAGAACACTGTGGCTTCACT			
F: CGAGAGTCTTTCTGACCTGTTC	9	60°C	398 bp
R: AAGGTCACCTTTGCAACAGC			
F: AGGCATGTCACTAATCTGGTCCAA	10	60°C	638 bp
R:GGGACTTCAGGGAAGATTCTGGTA			
F: GGGGTTCTCACATACATTTAAGCA	11	65°C	920 bp
R: GAGCTGTTGAACATGCCTGG			
F: ACTTCATGGGAGCCACCTCTAGAT	11	60°C	853 bp
R: AGACAGGTTGGTTGGTTGGTTGTG			

Blood samples of 39 healthy dogs were collected and DNA extraction was performed as previously reported [26]. Around 30 ng of genomic DNA were used in PCR reaction to amplify TET2 exon 11 and the products obtained were subsequently sequenced as described above.

Sequences were analyzed and aligned by using the SeqScape software v3.0 (Life Technologies, Carlsbad, USA) and identity percentage between dog protein sequences and mouse, rat, dog and cat were assessed through BLAST (https://blast.ncbi.nlm.nih.gov/Blast).

### Statistical analysis

To evaluate the possible relationship between the presence of glutamine repetitions in canine TET2 exon 11 and c-KIT mutations or the tendency to develop MCT, a Pearson χ^2^ correlation test was performed by GraphPad Prism version 5.00 for Windows (GraphPad Software, San Diego, USA). A value of P< 0.05 was considered significant.

## Results

### Gene sequences are conserved among canine and human species

In myeloproliferative disorders and particularly in SM, the majority of the genes considered in this study possess hot spot sites for mutations; therefore, in the first part of the study, a comparison between human and canine genomic sequences was performed to verify the potential conservation of the same mutations sites in dog and, subsequently, their localization. To give a general overview, the percentages of protein sequence identities in target genes between the canine and the other most commonly studied species (human, cat, mouse and rat) are reported in Table 2. In general, a high degree of conservation was noticed among them and, for our purposes, the CanFam3.1 genome sequence proved to be definitely complete and reliable.

**Table 2:** List of target genes and percentage of protein sequence identity between dog and other reference species (*Homo sapiens*, *Felis catus*, *Mus musculus*, *Rattus norvegicus*).

**Gene**	**Human**	**Cat**	**Mouse**	**Rat**
TET2	84%	91%	58%	60%
IDH1	97%	99%	95%	96%
IDH2	96%	99%	97%	96%
NRAS	100%	100%	100%	100%
KRAS	99%	97%	96%	96%
SF3B1	100%	100%	100%	100%
SRSF2	100%	NA	100%	100%

NA: sequence not available in the databases.

The amino acids residues considered hot spot sites for mutations in humans as R132 for IDH1, R140 and R170 for IDH2, G12 and Q61 for NRAS and G12 for KRAS were recognized in dog. Since in humans TET2 mutations occur almost all over the sequence, all the corresponding canine coding exons were amplified; the two sequences shared the 84% of amino acid identity (Table 2). On the other hand, the canine SRSF2 partial sequence obtained in this study differed, either in exons and introns, from NCBI release. Anyway, the analog of human hot spot site (P95) was conserved also in dog. The updated partial sequence was submitted to NCBI with the following accession number: KT072629.

### Target gene mutational status in MCTs samples

All genes were successfully amplified in all the 75 samples except for SRSF2, that was amplifiable in only 37 samples cause of its complexity and GC-richness.

In our MCT cohort, surprisingly, no mutations were ever found analyzing sequencing results of IDH1, IDH2, NRAS, KRAS, SF3B1 and SRSF2 genes (data not shown).

Among samples, some intronic variants not related with splicing sites were detected in the target genes. These alterations, with the relative allelic frequencies and population distribution, are collected in Table 3.

**Table 3:** List of genetic variations grouped for gene, relative population frequency and allelic frequencies in the MCT cohort of samples.

Gene	Intron/Exon	Variation	Population Frequency	Allelic frequencies
**TET2**	exon 3	c.732G>A p. =	3/75 (4%)	G: 0.98, A: 0.02
**TET2**	exon 3	c. 2315G>A: p.Gly772Asp	4/75 (5.3%)	G: 0.97, A: 0.03
**TET2**	intron 3	c.3439+75del	6/75 (8%)	T: 0.96, delT: 0.04
**TET2**	intron 8	c.4075-38del	5/75 (6.67%)	T: 0.97, delT: 0.03
**TET2**	intron 10	c.4212+63_4212+65insCAG	62/75 (82.7%)	WT: 0.31, insCAG: 0.69
**TET2**	intron 10	c.4568-65C>T	6/75 (8%)	C: 0.95, T: 0.05
**TET2**	exon 11	c.4914T>C:p =	58/75 (77.3%)	T: 0.41, C: 0.59
**TET2**	exon 11	c.5213A>G: p.Asn1728Ser	57/75 (76%)	G: 0.41, A: 0.59
**TET2**	exon 11	c.5278G>A: p.Ala1760Thr	1/75 (1.33%)	G: 0.01, A: 0.99
**IDH1**	intron 2	c. 292+37T>C	3/75 (4%)	T: 0.98, C: 0.02
**NRAS**	intron 2	c. 290+44C>T	18/75 (24%)	C: 0.87, T: 0.13
**KRAS**	intron 1	c. 93+104A>T	8/75 (10.67%)	A: 0.95, T: 0.05
**KRAS**	intron 1	c. 93+139T>C	8/75 (10.67%)	T: 0.95, C: 0.05
**SRSF2**	intron 1	362+59_362+62dup	21/37 (58%)	WT: 0.64, Dup: 0.36

### TET2 mutational status in dog MCT

In canine TET2 only two samples evidenced the presence of mutation in their coding sequence: one sample showed a heterozygous non-synonymous substitution (c.491A>G: p. Asp164Gly) in exon 3 while another sample was homozygous for a complete codon deletion (c.2226-2228del: p. His742del) always in exon 3. Both MCTs were histologically classified as Patnaik grade II. As regards to *c-KIT* mutations, the former had a wild-type sequence, while the second one had an internal tandem duplication occurring in exon 11 (ITD^572-583^). Since the frequency of TET2 mutations was low (2.7%), a correlation between TET2 and c-KIT mutational status and/or MCT histological grading, was not possible.

Also for TET2 gene, some intronic variants not related with splicing sites, and single nucleotide polymorphisms (SNPs) in the coding sequence were detected in the samples. These alterations, with the relative allelic frequencies and population distribution, are collected in Table 3.

**Fig 1:**
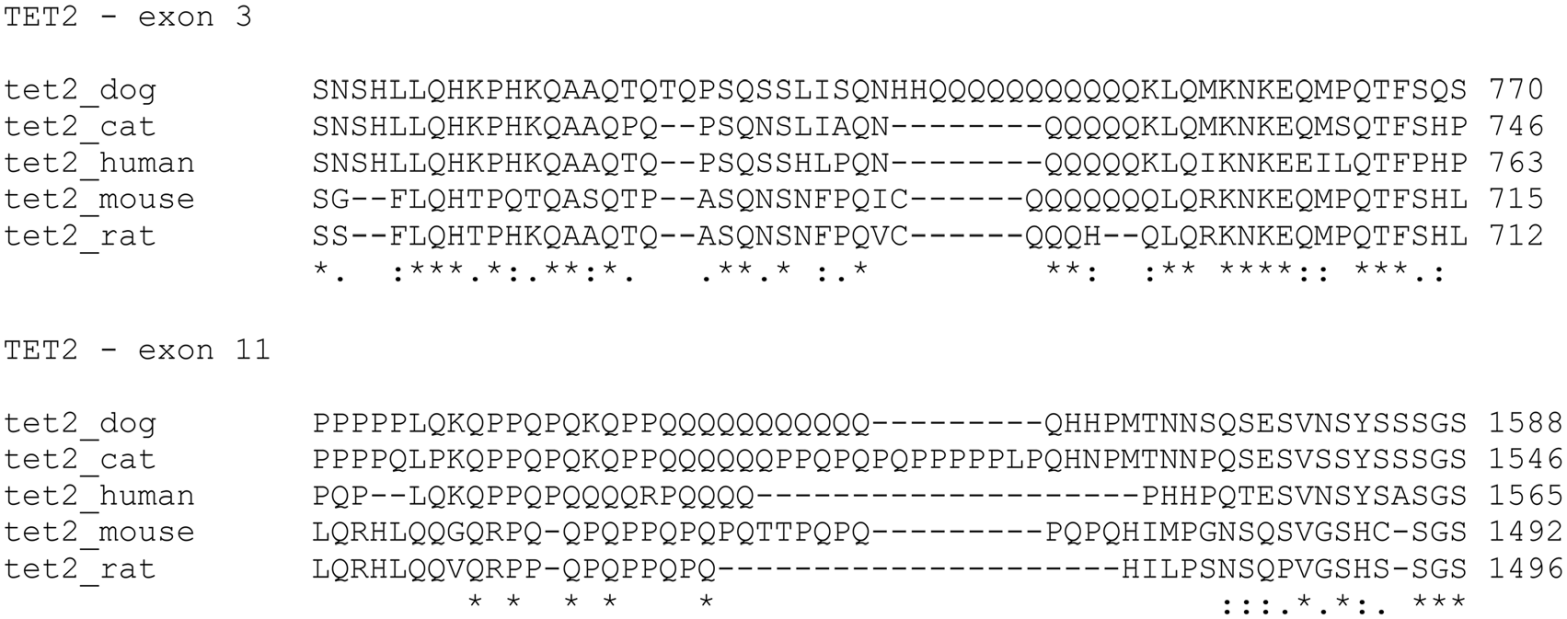
Sequence alignment between dog, cat, human, mouse and rat specific glutamine-rich regions located in exon 3 and 11 of TET2 gene. The image was obtained using the tool ClustalW2 (http://www.ebi.ac.uk/Tools/msa/clustalw2/).

Deepening in sequence analysis, other genetic variations were detected, in two specific regions of the gene. The protein database Uniprot (http://www.uniprot.org/) recognized them as polyglutamine rich-regions because they are rich in glutamine residues: one is located in exon 3 and another one in exon 11. The alignment of canine sequence with the same human, cat, mouse and rat sequence portion showed that poly-glutamines residues were quite conserved among species and dog possessed the highest number of glutamine repetitions (Fig 1). In details, in canine exon 3, 65 out of 75 MCTs (86,66%) showed the deletion of glutamine 753 (c. 2250_2252del; p.Gln753del). In exon 11, different rearrangements in the number of glutamine repetitions were detected among samples. All variations observed in our cohort of samples with relative frequencies in the group are listed in Table 4. Performing a Pearson χ^2^ analysis between the number of glutamine repetitions in each sample and the presence of c-KIT mutations, no statistical correlations were evident (P = 0.3427). Furthermore, the number of glutamine repetitions in exon 3 and 11 did not correlate with the histologic grade (Fisher exact test, P = 0.5808 and Pearson χ^2^, P = 0.2308, respectively).

**Table 4:** List of genetic variations detected in the glutamine rich region of TET2 exon 11 with relative population frequency and total glutamine residues number in the 75 MCT samples.

Variation	Population Frequency	Glutamine repetitions
Wild-type sequence	59/75	12
c. 4682insGCA; p. 1562insQ	5/75	13
c.4686_4697del; p. 1564_1567del	4/75	8
c.4686_4694del; p. 1564_1566del	3/75	9
c.4698_4700del; p.1568del	4/75	11

**Fig 2:**
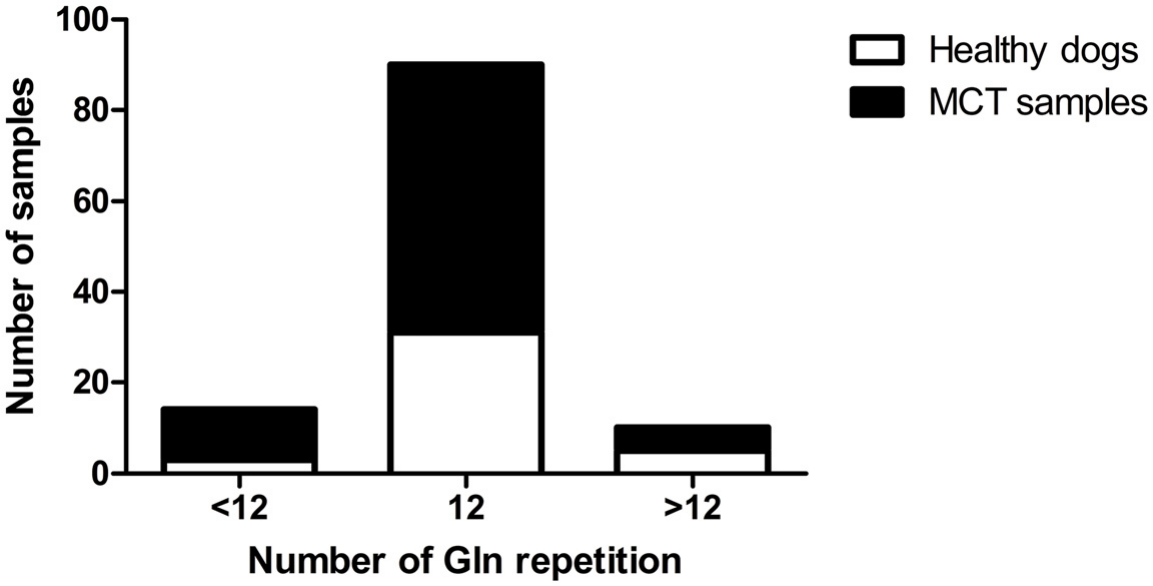
Association between the number of glutamine (Gln) repetitions and healthy/pathologic state in 114 canine blood and MCT samples. Pearson χ^2^ test (p = 0.3454; not significant).

Afterwards, to better understand if these sequence rearrangements might have a correlation with the onset of the disease, we screened the DNA from 39 healthy dogs for the same polyglutamine region in TET2 exon 11. Subsequently, considering as the wild-type phenotype the 12 glutamine repetitions presented in the reference NCBI sequence (XM_535678–4) we categorized all samples (healthy and pathologic) in three groups: samples that evidenced less than 12 glutamines (Q<12), wild-type dogs and samples with more than 12 glutamines (Q>12). From the contingency table and the Pearson χ^2^ test, no significant correlation emerged between the number of glutamine repetitions and the risk to develop mast cell tumor (Fig 2).

## Discussion

Mast cells (MCs) neoplastic disorders occur in both canine and human species sharing many but not all biological and clinical features. Spontaneous MCT has been proposed as a model to study biological and therapeutic approach for human neoplastic MCs diseases, i. e. mastocytosis [28]. At the same time, due to the implications of c-KIT aberrations in the development of MCs tumors, canine MCT could represent a useful model to study human c-KIT driven malignancies and TKIs, targeting c-kit. Therefore, comparative studies of MCs disorders may represent an opportunity to improve our knowledge on both mastocytosis and c-KIT driven tumors for diagnosis in case of c-KIT wild type state and/or with the aim to develop novel treatment options that can be translated in human patients.

In this respect, starting from a list of genes that showed recurrent somatic mutations in human myeloproliferative diseases and mastocytosis, we screened a cohort of 75 canine MCTs for hot-spot mutations sites.

No mutations were identified in IDH1 and IDH2 genes in our cohort of MCTs while, in SM, IDH2 mutations occurred for 6.9% of cases [11]. To the best of our knowledge, only one study has been published in dog [29] where no mutations in both these genes were found in canine gliomas. Considering the high percentage of mutations in the human analog tumor (~ 80% in grades II-III) these results were surprising and might suggest a minor role of these genes in the pathogenesis of canine gliomas and MCT.

In SM, two genes involved in spliceosome machinery, SRSF2 and SF3B1, showed a mutation occurrence of 24% and 5.6% respectively [11]. However, no mutations were detected in canine MCT. No data about the relevance of these genes and their mutational status in canine oncology are actually available; therefore, present results, are the first data ever published so far.

On the other hand, more information are available about NRAS and KRAS, in dog cancer. Present results showing the absence of mutations obtained in our MCTs samples are consistent with a number of previously published studies in which RAS mutations have been shown to be extremely rare in the most common types of canine tumor such as mammary tumors, soft-tissue tumors (included MCTs), melanomas and lymphoproliferative disorders [30,31]. In contrast, higher mutational frequencies of RAS genes have been obtained in human lung, pancreatic, gastrointestinal, brain and liver tumor, in acute myelogenous leukemia, in follicular and undifferentiated papillary thyroid tumors [32]. Therefore and likewise to IDH1/2 we might hypothesized that RAS mutations do not play a major role in the pathogenesis of canine MCT and this supposition is in line with previously published data [30].

About TET2, the high percentage of mutations found in aggressive form of human mastocytosis (20.8%) was not confirmed in canine MCT (2.7%). Moreover, the typical frame-shift mutations observed in humans and coding for a truncated protein with consequently loss of function, was never observed. These results surprised the authors and, until this moment, represented the first attempt, in veterinary medicine, to investigate the role of TET2 mutations in a canine tumor since no information are available in previous published studies.

Noteworthy, a frequent rearrangement was observed in a glutamine-rich region of TET2 exon 11, resulting in variations of the number of glutamine repetitions (from 8 to 13) among cases. Very little information are actually published in human oncology about a possible relationship between length of polyglutamine regions in some genes and the risk to develop cancer. The number of CAG repetitions in androgen receptor seems to be correlated with the risk of occurrence of prostate cancer; furthermore polymorphisms in glutamine regions of nuclear receptor coactivator 3 (NCOA3 also known as AIB1) seems to play a role in the susceptibility of some type of breast cancer [33–35]. The sequencing analysis conducted in a little group of healthy dogs and matched statistically with results of MCT samples did not reveal any significant relationship between number of glutamine repetitions and the risk of MCT development.

In conclusion, this preliminary study aimed to investigate, in canine MCT, the mutational status of genes known to be involved in human myeloproliferative disorders. The study was undertaken in a relatively small cohort of canine samples, and only human analogue hot-spot sites for mutation were took into consideration. Further investigations are needed to better characterize the pathogenic pathways involved in both diseases. Among these ones, to sequence the entire IDH1, IDH2, NRAS, KRAS, SRSF2 and SF3B1 genes and, subsequently, to analyze interesting genes that were excluded from this preliminary study (i. e. Additional Sex Combs Like 1 alias ASXL1, Janus Kinase 2 alias JAK2). Clearly, the advent of deep sequencing methods might be more useful in this sense, giving a more wide observation on genome modifications and allowing the identification of new hot-spot mutation sites in canine genes. This approach will permit to clarify the possible value of canine MCT as a comparative animal model for human SM.
